# Right ventricular volumes and function in thalassemia major patients in the absence of myocardial iron overload

**DOI:** 10.1186/1532-429X-12-24

**Published:** 2010-04-23

**Authors:** John-Paul Carpenter, Francisco Alpendurada, Monica Deac, Alicia Maceira, Maciej Garbowski, Paul Kirk, J Malcolm Walker, John B Porter, Farrukh Shah, Winston Banya, Taigang He, Gillian C Smith, Dudley J Pennell

**Affiliations:** 1 Cardiovascular MR Unit, Royal Brompton Hospital, London, London, UK; 2 National Heart and Lung Institute, Imperial College London, London, UK; 3 Cardiac Imaging Unit, ERESA, Valencia, Spain; 4 Department of Haematology, University College London, London, UK; 5 Department of Haematology, Whittington Hospital, London, UK

## Abstract

**Aim:**

We aimed to define reference ranges for right ventricular (RV) volumes, ejection fraction (EF) in thalassemia major patients (TM) without myocardial iron overload.

**Methods and results:**

RV volumes, EF and mass were measured in 80 TM patients who had no myocardial iron overload (myocardial T2* > 20 ms by cardiovascular magnetic resonance). All patients were receiving deferoxamine chelation and none had evidence of pulmonary hypertension or other cardiovascular comorbidity. Forty age and sex matched healthy non-anemic volunteers acted as controls. The mean RV EF was higher in TM patients than controls (males 66.2 ± 4.1% vs 61.6 ± 6%, p = 0.0009; females 66.3 ± 5.1% vs 62.6 ± 6.4%, p = 0.017), which yielded a raised lower threshold of normality for RV EF in TM patients (males 58.0% vs 50.0% and females 56.4% vs 50.1%). RV end-diastolic volume index was higher in male TM patients (mean 98.1 ± 17.3 mL vs 88.4 ± 11.2 mL/m2, p = 0.027), with a higher upper limit (132 vs 110 mL/m2) but this difference was of borderline significance for females (mean 86.5 ± 13.6 mL vs 80.3 ± 12.8 mL/m2, p = 0.09, with upper limit of 113 vs 105 mL/m2). The cardiac index was raised in TM patients (males 4.8 ± 1.0 L/min vs 3.4 ± 0.7 L/min, p < 0.0001; females 4.5 ± 0.8 L/min vs 3.2 ± 0.8 L/min, p < 0.0001). No differences in RV mass index were identified.

**Conclusion:**

The normal ranges for functional RV parameters in TM patients with no evidence of myocardial iron overload differ from healthy non-anemic controls. The new reference RV ranges are important for determining the functional effects of myocardial iron overload in TM patients.

## Introduction

Patients with beta-thalassemia major (TM) have a severe hereditary anemia which requires lifelong transfusions to prolong survival and allow normal development [1]. Due to the absence of an effective physiological excretory pathway in humans, the unwanted consequence of these blood transfusions is iron overload, predominantly affecting the heart, liver and endocrine organs. Despite recent improvements in patient care, iron overload cardiomyopathy remains a leading cause of death in TM patients in many centers [2,3]. The early detection of iron-induced cardiac toxicity therefore forms a key component of clinical management. The assessment of cardiac iron loading can be performed directly by measurement of myocardial T2* (explicit myocardial iron assessment) or indirectly by the assessment of ventricular volumes and function (examination of effects of myocardial iron on cardiac function).

Previously published data have shown that indices of the left ventricle (LV) such as volumes and ejection fraction (EF) differ in non-cardiac iron loaded TM patients from healthy non-anemic controls, most likely due to chronically increased cardiac output related to the anemia [4,5]. These differences in the normal range of expected values affect the interpretation of measures of ventricular function from echocardiography and cardiovascular magnetic resonance (CMR). This is important with regard to the early detection of impaired EF because the use of inappropriate reference values may mask the diagnosis of underlying iron-overload cardiomyopathy and this can result in delayed treatment or a preventable episode of heart failure, which places the patient at high hazard [6]. Conversely, an apparently dilated heart in a TM patient may be within normal limits for the non-iron overloaded TM population. Although it has been shown that both RV and LV EF are reduced by iron loading [7,8], the normal ranges for RV parameters and function in TM patients who have no evidence of cardiac iron loading are unknown. The aim of this study therefore was to define the normal reference ranges for RV volumes, ejection fraction and mass in non-iron overloaded transfusion dependent TM patients in comparison with non-anemic healthy controls. CMR was used for this assessment as it is regarded as the gold-standard technique for measurement of both LV and RV volumes and function [9-11], and CMR can also measure myocardial iron loading using myocardial T2*.

## Methods

### Study population

We performed a retrospective analysis of patients with beta-thalassemia major who were referred for their firstmyocardial T2* scan from 21 UK hematology centres. All patients were regularly transfused (every 3-4 weeks) to maintain pre-transfusion hemoglobin levels of 9-10 g/dl and all had received iron chelation therapy from an early age or from the mid-to-late 1970s if born before this time. To remove any possible effects of different iron chelating agents, only patients taking deferoxamine as a single iron chelator were included. None of the patients had received treatment with either of the oral chelating agents (deferiprone or deferasirox). Forty male and forty female patients over the age of 18 years who had no myocardial iron loading (defined as having cardiac T2* > 20 ms) and no history of any known cardiovascular pathology were identified from the initial target population of 323 patients. The cut-off value for normal T2* was based on the lower limit of normal observed in a cohort of healthy volunteers [7]. Patients with evidence of pulmonary hypertension (defined as tricuspid regurgitant velocity > 3.0 m/s at rest by transthoracic echocardiography) were excluded. Forty age and sex matched healthy non-anemic volunteers formed a control population for comparison. All control subjects were healthy, asymptomatic volunteers with no cardiovascular risk factors or history of cardiac disease. Each had a normal 12 lead electrocardiogram and no abnormal signs on physical examination. This study was approved by the local NHS Research Ethics Committee. Written informed consent was obtained from all of the volunteers. For the TM patients, the Ethics Committee granted permission for review of clinical and scan data, waiver of informed consent and anonymous publication.

### Cardiovascular magnetic resonance

All scans were performed using a 1.5T Sonata scanner (Siemens Medical Systems, Erlangen, Germany). After routine localizer images, each scan comprised of a contiguous set of breath-hold steady state free precession (SSFP) short-axis cines at 10 mm intervals from base to apex (7 mm slice thickness with 3 mm gap) using standardised techniques [5,12]. An ECG gated breath-hold bright blood multi-echo sequence was also used to acquire a single short axis mid-ventricular slice for the measurement of myocardial T2* (a gradient echo sequence acquired immediately after the R-wave trigger with flip angle of 35°, matrix of 128 × 256 pixels, field of view (FOV) 40 cm, bandwidth of 810 Hz per pixel and repetition time (TR) of 20 ms between each radiofrequency (RF) pulse). This sequence generated a series of images with a range of equally spaced echo times (TE = 2.6-16.7 ms) [13].

### CMR analysis

**Figure 1:**
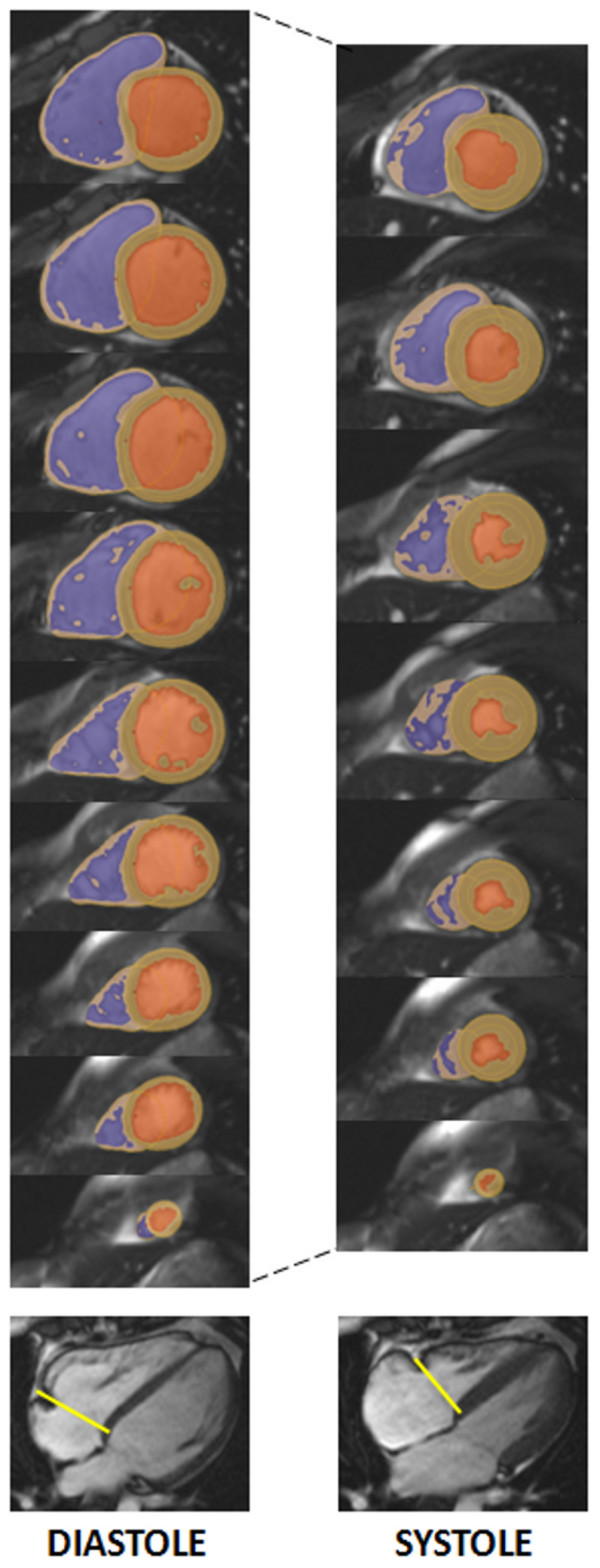
**Calculation of RV parameters**. Delineation of right ventricular endocardial and epicardial borders using semi-automated software, and summing up over all contiguous slices covering the right ventricle allows the calculation of all volume, mass and functional parameters. Representative images are shown for end-diastole and end-systole together with tricuspid valve plane tracking (indicated by the yellow line on the four-chamber view). The RV blood pool is shown in blue, the LV blood pool in orange and the myocardium in beige.

Right ventricular volumes and mass were measured from the SSFP cines as previously described [12,14], using CMRtools (Cardiovascular Imaging Solutions, London). This involved tracing the endocardial and epicardial borders at end-diastole and end-systole with semi-automated thresholding to delineate the blood pool (figure 1). RV trabeculations were excluded from the blood pool volume but included in the RV mass calculation. The tricuspid valve plane was tracked in both systole and diastole to ensure that any blood signal from the right atrium was excluded from the ventricular volume calculation. Any of the blood pool signal above the pulmonary valve was also excluded from the ventricular volume using the endocardial contour definitions. Cardiac output was calculated from the product of right ventricular stroke volume and the mean heart rate recorded at the time of the CMR scan. RV parameters were indexed to body surface area (BSA) which was derived using the Mosteller formula [12,15]. Myocardial T2* was measured from a single full thickness region of interest in the septum of the mid-ventricular slice using semi-automated software (Thalassemia-tools, Cardiovascular Imaging Solutions, London, UK). For the analysis of T2*, mean signal intensity was plotted against the echo time for each image in the series. The T2* value was calculated as previously described from the resulting exponential decay curve after truncating the curve to correct for background noise [16].

### Hemoglobin measurements

Pre-transfusion hemoglobin measurements were compared with right ventricular parameters. Where the interval between the hemoglobin measurement and the index CMR scan exceeded 1 week, patients were excluded from this part of the analysis.

### Statistical analysis

All continuous parameters were found to be normally distributed and are therefore presented as mean ± standard deviation (SD). An unpaired two-tailed t-test was used to compare TM patients with the healthy non-anemic volunteers. Separate analysis was performed for males and females due to known gender-specific differences for left and right ventricular parameters. Pearson correlation was used to compare hemoglobin measurements with the RV volumes and function measurements. Statistical significance was set at p < 0.05. All statistical analysis was performed using Stata 10.1 software (StataCorp, Texas, USA).

## Results

### Patient population

A summary of the demographics for the patients and the control population is given in table 1. Both groups were well matched for age and sex. The body mass index was equivalent in females but was slightly higher in the male control population than the TM patients. However, both male and female TM patients had significantly lower weight, height and body surface area than the non-anemic controls. Resting heart rate in TM patients was also significantly higher than in the healthy controls.

**Table 1:** Demographics for TM patients and controls.

	TM patients Mean ± SD	Controls Mean ± SD	P value
Males			
Age (years)	30 ± 8	30 ± 5	0.94
Height (m)	1.65 ± 0.1	1.80 ± 0.09	<0.0001
Weight (kg)	59.1 ± 8.9	75.8 ± 9.9	<0.0001
BMI (kg/m^2^)	21.9 ± 3.1	23.6 ± 3.1	0.048
BSA (m^2^)	1.64 ± 0.16	1.94 ± 0.15	<0.0001
Heart rate (min^-1^)	72.7 ± 10.4	64.0 ± 9.5	0.0024

Females			
Age (years)	30 ± 8	30 ± 5	0.88
Height (m)	1.54 ± 8.9	1.67 ± 9.2	<0.0001
Weight (kg)	53.3 ± 10.1	61.4 ± 11.3	0.0064
BMI (kg/m^2^)	22.5 ± 4.5	22.0 ± 2.8	0.68
BSA (m^2^)	1.50 ± 0.15	1.68 ± 0.18	0.0001
Heart rate (min^-1^)	78.8 ± 10.1	64.8 ± 13.6	<0.0001

BSA -- body surface area, BMI -- body mass index.

### Right ventricular parameters

**Figure 2:**
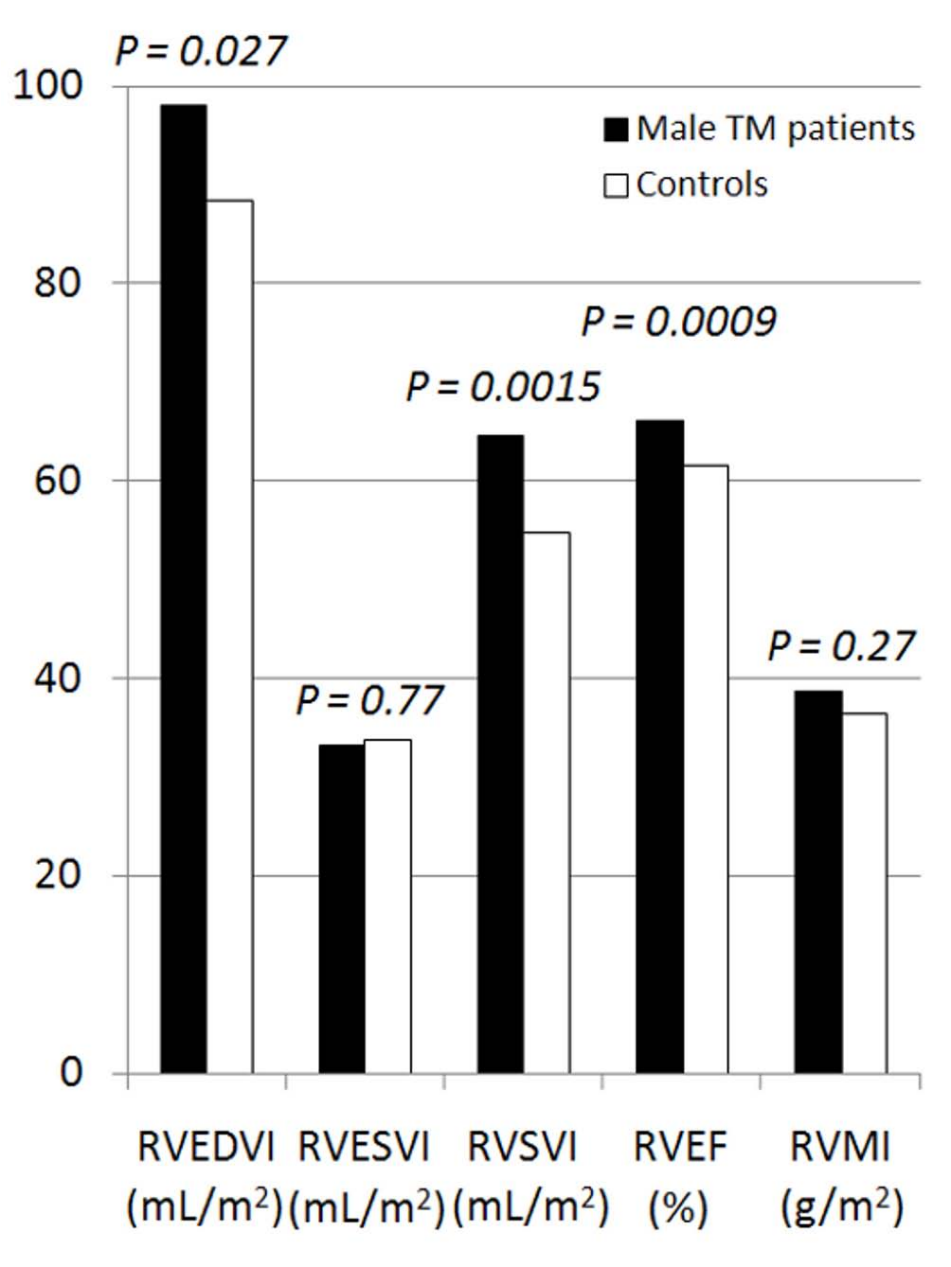
**Right ventricular volumes and ejection fraction in male TM patients and controls**. RVEDVI = right ventricular end-diastolic volume index, RVESVI = right ventricular end-systolic volume index, RVSVI = right ventricular stroke volume index, RVEF = right ventricular ejection fraction, RVMI = right ventricular mass index.

**Figure 3:**
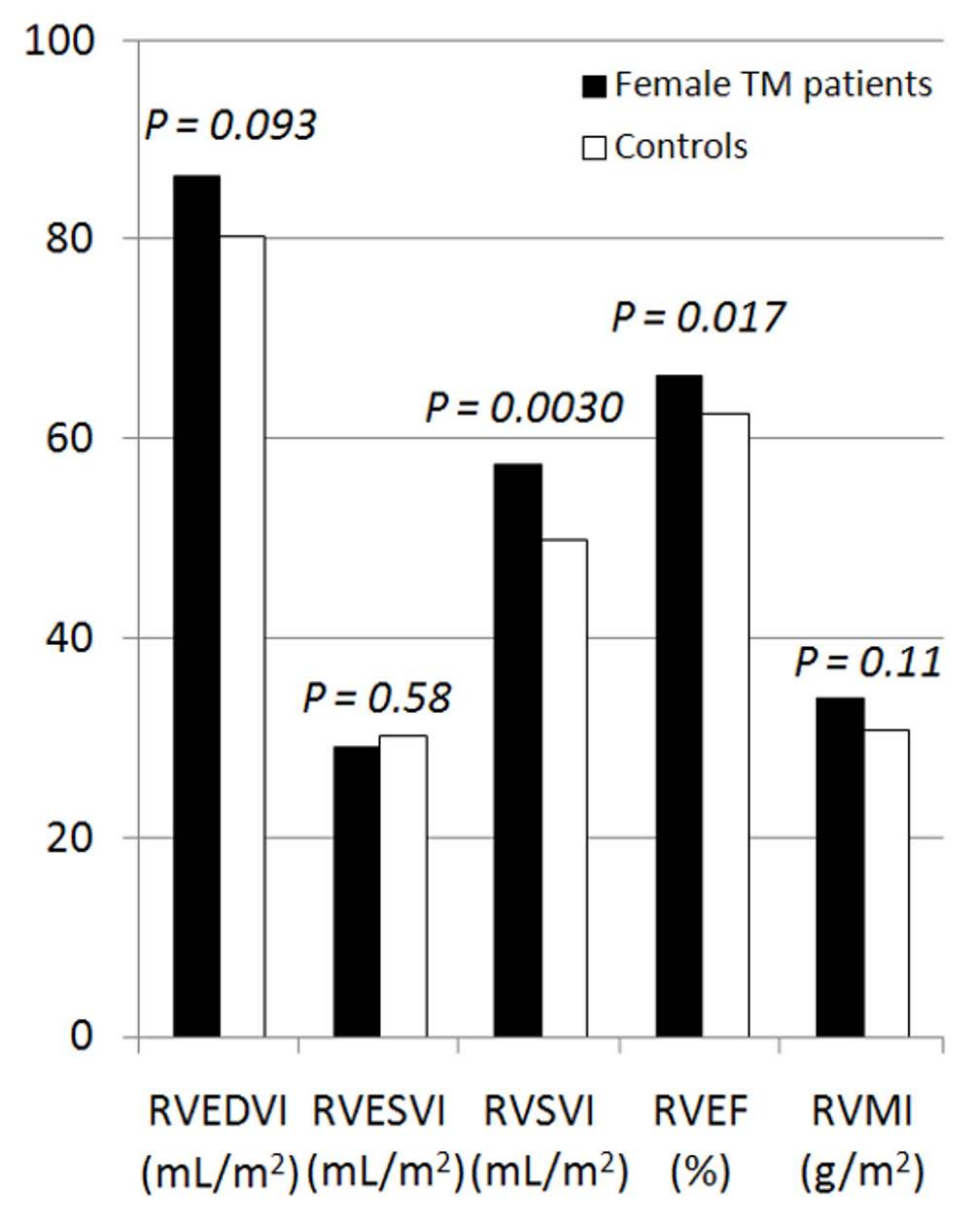
**Right ventricular volumes and ejection fraction in female TM patients and controls**. RVEDVI = right ventricular end-diastolic volume index, RVESVI = right ventricular end-systolic volume index, RVSVI = right ventricular stroke volume index, RVEF = right ventricular ejection fraction, RVMI = right ventricular mass index.

The right ventricular parameters are detailed in table 2, and represented graphically in figures 2 and 3. The mean and upper limit of end-diastolic RV volume were higher in TM patients than controls for males, but this was borderline significant for females (p = 0.027 for males, p = 0.093 for females). RV stroke volume and RV ejection fraction were higher in TM patients for both males (p = 0.0015 for stroke volume, p = 0.0009 for RV EF) and females (p = 0.0030 for stroke volume, p = 0.017 for RV EF). The lower limit of RV EF was higher in TM patients (males 58.0% vs 50.0%, females 56.4% vs 50.1%). The cardiac output was higher in the TM cohort than controls (p = 0.014 for males, p = 0.0033 for females) and this finding was confirmed when cardiac output was indexed for BSA (cardiac index, p < 0.0001). No significant difference was found however between TM patients and controls for either RV end-systolic volume index or RV mass index (p = 0.11 to 0.77).

**Table 2:** Right ventricular parameters for males and females.

	TM patients (mean ± SD) [95% CI]	Controls (mean ± SD) [95% CI]	P value
Males			
RVEDVI (mL/m^2^)	98.1 ± 17.3 [64.2 -- 132.0]	88.4 ± 11.2 [66.5 -- 110.4]	0.027
RVESVI (mL/m^2^)	33.2 ± 8.0 [17.5 -- 48.8]	33.8 ± 5.0 [24.0 -- 43.7]	0.77
RVSVI (mL/m^2^)	64.7 ± 11.2 [42.7 -- 86.6]	54.7 ± 10.3 [34.6 -- 74.8]	0.0015
RVEF (%)	66.2 ± 4.1 [58.0 -- 74.3]	61.6 ± 6.0 [50.0 -- 73.3]	0.0009
RVMI (g/m^2^)	38.8 ± 7.9 [23.3 -- 54.4]	36.4 ± 7.8 [21.1 -- 51.7]	0.27
CO (L/min)	7.9 ± 1.9 [4.16 -- 11.5]	6.6 ± 1.6 [3.4 -- 9.8]	0.014
CI (L/min/m^2^)	4.8 ± 1.0 [2.9 -- 6.7]	3.4 ± 0.7 [2.0 -- 4.8]	<0.0001

Females			
RVEDVI (mL/m^2^)	86.5 ± 13.6 [59.8 -- 113.2]	80.3 ± 12.8 [55.3 -- 105.3]	0.093
RVESVI (mL/m^2^)	29.2 ± 7.2 [15.1 -- 43.2]	30.3 ± 8.6 [13.5 -- 47.1]	0.58
RVSVI (mL/m^2^)	57.4 ± 9.2 [39.4 -- 75.5]	50.0 ± 7.8 [34.7 -- 65.2]	0.0030
RVEF (%)	66.3 ± 5.1 [56.4 -- 76.2]	62.6 ± 6.4 [50.1 -- 75.0]	0.017
RVMI (g/m^2^)	34.0 ± 7.7 [18.8 -- 49.1]	30.8 ± 5.3 [20.5 -- 41.1]	0.11
CO (L/min)	6.8 ± 1.7 [3.5 -- 10.1]	5.5 ± 1.5 [2.5 -- 8.4]	0.0033
CI (L/min/m^2^)	4.5 ± 0.8 [2.9 -- 6.2]	3.2 ± 0.8 [1.7 -- 4.8]	<0.0001

All values are quoted as mean ± SD with 95% confidence intervals in square brackets. The table is divided into TM patients with no evidence of cardiac iron overload and age-matched healthy controls.

RVEDVI = right ventricular end-diastolic volume index, RVESVI = right ventricular end-systolic volume index, RVSVI = right ventricular stroke volume index, RVEF = right ventricular ejection fraction, RVMI = right ventricular mass index, CO = cardiac output, CI = cardiac index

### Correlation with hemoglobin levels

Hemoglobin results which coincided with CMR scans (blood tests within one week of the scan) were obtained in 59% of the patients investigated. There was no difference in any of the RV parameters between those patients with hemoglobin results and those in whom the results were unavailable. Mean hemoglobin level was 9.7 ± 1.8 g/dL for males (n = 20) and 10.5 ± 1.4 g/dL for females (n = 27). In the female TM patients, no significant correlations existed between hemoglobin concentration and any of the RV parameters. In male TM patients, an inverse correlation was found between cardiac index and hemoglobin (r = -0.47, p = 0.04). No other significant correlation was found.

## Discussion

Cardiac complications due to myocardial siderosis remain a serious problem for TM patients. Until recently, more than 50% of TM patients died before the age of 35 from cardiac failure [17]. The monitoring of cardiac iron using T2* CMR has had a major impact on saving the lives of patients by identifying cardiac iron overload prior to the occurrence of heart failure which therefore allows tailored cardiac chelation [3]. However, cardiac T2* is not available in all centers and non-cardiac measures of iron loading are not satisfactory for assessing the risk of heart failure in comparison with cardiac T2* [18]. An indirect approach to assessment of cardiac iron loading is to measure cardiac volumes and function. Although the literature establishing the value of this approach is rather sparse [19], it has the merit that techniques for assessment of cardiac function such as echocardiography are widely available and its application is included in some clinical guidelines [20]. In favor of the use of functional heart measurements is the clear evidence of a correlation with cardiac iron loading that is not present for cross-sectional measures of blood iron (ferritin) or liver iron [7]. Any such approach however, requires that normal values for TM patients who do not have cardiac iron loading are established. Previous data has shown that left ventricular volumes and function in non-cardiac iron loaded TM patients are significantly different from healthy non-anemic controls [4], but there is no data on the normal values of RV volumes and function in non-cardiac iron loaded patients with TM. The right ventricle has consistently been underestimated as an important factor in heart disease and its power to predict adverse cardiac outcomes, which is independent and additional to LV function, has often been overlooked in the past. The balance has been addressed in a number of relatively recent studies of the RV in association with outcomes in heart failure syndromes related to dilated cardiomyopathy [21], chronic systolic dysfunction [22], and ischemia [23-25], and also in patients with congenital heart disease [26-28]. This suggests that RV function may be a significant contributor to the clinical manifestation of heart failure seen in myocardial iron overload. Therefore in this study, we evaluated RV parameters using CMR in a population of regularly transfused TM patients with no cardiac iron loading or pulmonary hypertension or other cardiac morbidity and have compared the findings to those of healthy non-anemic subjects to established reference ranges that would prove useful to assess the functional effects on the heart of iron overload. Many TM patients have growth retardation with short stature and low body weight. The direct comparison of raw RV indices between TM patients and a cohort of healthy non-anemic subjects therefore requires indexing the RV volumes to body surface area.

Our results show that compared with healthy non-anemic controls, TM patients have a higher RV stroke volume and heart rate, which results in a higher cardiac output. The RV EF is also increased mainly as a result of an increased end-diastolic volume. These results are similar to the observations of LV parameters in TM but the differences in RV parameters appear to be less pronounced than those found for the LV [4]. Our results stress that if functional measurements of the heart are to be made and used clinically to indirectly assess cardiac iron loading, then it is vital to use reference ranges from TM patients with no cardiac iron loading as presented from this study in order to prevent underdiagnosis of cardiac siderosis when using the EF, or its overdiagnosis when using the end-diastolic volume.

No correlation between hemoglobin level and RV parameters was identified in female TM patients but in males, there was an inverse correlation between hemoglobin and cardiac index. It is not evident why male and female patients differ but the result mirrors previous observations [4]. An inverse relationship between hemoglobin and cardiac index is predictable, and indicates a higher output state with a greater degree of anemia. There was no correlation between hemoglobin and RV EF for either sex, a finding supported by a previous study which found no difference in LV EF using radionuclide ventriculography both before and 24 hours after blood transfusion [19]. Pulmonary hypertension (which may depress RV function and cause right heart failure) has been described in thalassemia patients but although it is prominent in those with thalassemia intermedia, it is uncommon in well treated TM patients [29-31]. Not only have we purposely excluded patients with pulmonary hypertension from this study, but we also found no significant difference in RV mass between cases and controls, a sensitive and specific measure for the diagnosis of pulmonary hypertension using CMR [32]. Therefore we believe there is no confounding of our results from pulmonary hypertension.

The right ventricle has a complex anatomical structure in comparison to the LV. Whereas the LV is approximately circular in cross-section, the RV is crescentic, wrapping around the LV with separate inflow and outflow portions, the tricuspid and the pulmonary valves being physically separated by a muscular subpulmonary infundibulum. The RV is thin walled with many trabeculations and there is a moderator band of myocardial tissue towards the apex. All of these features create challenges for modeling RV volumes, making it more difficult to perform reliable measurements using standard echocardiographic techniques. CMR is able to overcome most of these issues and is currently considered the gold standard for the measurement of cardiac volumes and function [9], with well defined normalized values for the RV [12]. The relative accuracy of echocardiography in relation to CMR must therefore be considered when interpreting results of RV measurements in clinical practice.

There is previously published data regarding RV function in patients with established heart failure due to myocardial siderosis and other studies have reported RV parameters in TM patients across a wide range of iron loading [33-35]. However, our study focuses only on RV volumes and function in TM patients without evidence of cardiac iron loading.

### Limitations

We have restricted our investigation to CMR parameters of RV function and comparisons with RV measurements from other imaging modalities should be interpreted with caution. While RV EF is the most widely available method for assessing RV function, it may not adequately reflect RV contractility and other techniques for the assessment of the RV may provide additional insights. For the hemoglobin correlation, we only used results in a subset of the patients for which the time between the CMR scan and the hemoglobin estimation was less than 1 week. Subset analysis showed no significant differences in any of the RV parameters between patients with and those without hemoglobin results. We did not have reliable information regarding the date of the most recent transfusion prior to the CMR assessment in the TM cohort. While transfusion could potentially affect RV parameters including RV EF, there is only limited data regarding the effects of transfusion on ventricular function. For the LV, no significant difference in LV EF is observed between measurements taken before or 24 hours after blood transfusion [19].

## Conclusion

Our findings show that the normal ranges for RV parameters differ between TM patients without cardiac iron loading and normal, non-anemic controls. The lower limit of RVEF in TM patients without cardiac iron loading is significantly higher than the lower limit of the normal range in controls which could lead to under-diagnosis of iron-loading cardiomyopathy if this is not appreciated. It is important to use reference ranges which are specific to non-cardiac iron loaded TM patients when assessing cardiac volumes and function as a surrogate for cardiac iron loading.

## List of abbreviations

**BSA**: body surface area; **CMR**: Cardiovascular magnetic resonance; **ECG**: electrocardiograph; **EF**: ejection fraction; **FOV**: field of view; **Hz**: Hertz; **LV**: left ventricle; **NHS**: National Health Service (UK); **RV**: right ventricle; **RF**: radiofrequency; **SD**: standard deviation; **SSFP**: steady state free precession; **T**: Tesla; **TE**: echo time; **TM**: thalassemia major; **TR**: repetition time; T2*: T2 star; **UK**: United Kingdom; **USA**: United States of America.

## Competing interests

DJP is a consultant to Novartis, ApoPharma and Siemens, and is a director of Cardiovascular Imaging Solutions. DJP has received research support and speakers honoraria from Siemens, Novartis and ApoPharma. JPC has received speaker's honoraria from Swedish Orphan and ApoPharma. JBP has received research support from and has performed advisory board work for Novartis. GCS and TH have received consultancy fees from Novartis. The other authors report no conflict of interests.

## Authors' contributions

JPC and FA both participated equally in the study design, data acquisition, and drafting of the manuscript; MD acquired data and drafted the manuscript; AM, MG, PK, JMW, JBP, FS, TH and GCS acquired data; WB performed the statistical analysis; DJP conceived and co-designed the study, and is responsible for the final manuscript.
